# The patient’s relationship with the General Practitioner before and after Advance Care Planning: pre/post-implementation study

**DOI:** 10.1186/s12877-022-03256-4

**Published:** 2022-07-05

**Authors:** Annicka G. M. van der Plas, Julia E. A. P. Schellekens, Jolien J. Glaudemans, Bregje D. Onwuteaka-Philipsen

**Affiliations:** 1 Department of Public and Occupational Health, Amsterdam UMC, Location VU University Medical Center, PO Box 7057, 1007 MB Amsterdam, The Netherlands; 2 Expertise Center for Palliative Care, Amsterdam UMC, PO Box 7057, 1007 MB Amsterdam, The Netherlands; 3 Amsterdam UMC, Location AMC, Section of Medical Ethics, Department of General Practice, Amsterdam Public Health Research Institute, Amsterdam, The Netherlands

**Keywords:** Advance care planning, Advance directives, Communication, General practice, Health services for the aged, Physician–patient relations

## Abstract

**Background:**

General Practitioners (GPs) are central in the care of Dutch older people and in a good position to have Advance Care Planning (ACP) conversations. Interview studies reveal that the doctor-patient relationship is important when initiating ACP conversations and can also be influenced by ACP conversations. We aimed to examine the association between having an ACP conversation and the patient feeling the GP knows him or her and the patient trusting the GP and vice versa.

**Methods:**

Implementation of ACP in primary care was evaluated in a pre-and post design. Questionnaires before implementation of ACP and 14 months later were sent to patients aged 75 years or older within 10 GP-practices and 2 care homes. Multivariable logistic regression was used to model the relationship between ACP conversations during implementation and the patient-GP relationship before implementation. Odds ratios were adjusted for potential confounders. Generalized ordered logistic regression was used to model the relationship between the changes in patient-GP relationship before and after implementation and ACP conversations during implementation.

**Results:**

Four hundred fifty-eight patients filled out the pre- and post-test questionnaire. There was no association between the GP knowing the patient and trust in the pre-test and having an ACP conversation during the implementation. For people who had had an ACP conversation at the end of the implementation period their trust remained more often the same or was higher after implementation (trust to provide good care OR 2.93; trust to follow their wishes OR 2.59), compared to patients who did not have an ACP conversation. A reduction in trust was less likely to happen to patients who had an ACP conversation compared to patients who did not have an ACP conversation.

**Conclusions:**

Although we have not found evidence for trust as a prerequisite for ACP conversations, this paper shows that ACP conversations can be beneficial for the doctor—patient relationship.

**Supplementary Information:**

The online version contains supplementary material available at 10.1186/s12877-022-03256-4.

## Background

Advance care planning (ACP) enables individuals to define goals and preferences for future medical treatment and care, to discuss these goals and preferences with family and health-care providers, and to record and review these preferences if appropriate [1]. Current literature is moving away from Advance Directives as focal point in planning end-of-life care and stressing the importance of the communication process: “Interventions that ignore the human connection will miss an important piece” [2, 3]. However, the association between having an ACP conversation and the patient’s relationship with healthcare providers before and after the conversation is unclear.

The doctor-patient relationship involves longitudinal care and consultation during which knowledge, trust, loyalty, and regard (or the lack thereof) are experienced [4]. Trust is an important aspect of the doctor-patient relationship. Trust in healthcare providers can be described as the optimistic acceptance of a vulnerable situation in which the truster believes the trustee will care for the truster’s interests [5]. There are qualitative studies that examine ACP and the relationship between the healthcare provider and patient. In most studies, the relationship, and especially trust, is mentioned as an important starting point for ACP [6–9]. There are also indications that ACP may affect the patient-doctor relationship. Patients with end stage renal disease saw ACP as an important element in a trusting patient-doctor relationship and felt this relationship was threatened when their concerns regarding severity of their disease, fears about dialysis and the future were not addressed by the doctor [10]. In a scoping review of outcomes of ACP in randomised controlled trials, the quality of patient-clinician conversations was positively evaluated in 7 out of 7 reported studies [11].

In Dutch primary care General Practitioners (GPs) are central in the care for older people. GPs are likely to have good clinical and contextual knowledge of their patients because of their long-term relationship [12]. Patients want to have ACP discussions with their GPs and think it is important for GPs to initiate those discussions [13]. As in studies with other healthcare providers, most studies regarding GPs and ACP are qualitative and mention the relationship as starting point. Healthcare providers in a study on ACP with older persons in care homes and in the community, expressed the opinion that interpersonal relationships were a fundamental requisite to end-of-life care discussions [14]. In a study on ACP conversations between GPs and people with dementia, all participants (GPs, patients, their carers) of the study agreed that it is important that the GP knows the person with dementia personally, is empathic, supportive and provides information respectfully [15]. In a recent study by Glaudemans [16], a lack in trust or negative previous experiences with ACP with a GP or nurse could be a reason for older people to be less open to ACP conversations. At the same time, older people who did engage in ACP felt they could trust their GP or nurse more after the ACP conversation. They were positive about the attention they received during these conversations, felt heard and more at ease.

To our knowledge no quantitative research has been done to further examine the association between having an ACP conversation and the patient’s relationship with the GP before and after the conversation. In this planned secondary analysis using data from a study on the implementation of ACP (see Table 1) [17], we address the following questions:

1. Is there an association between the patient’s relationship (the patient feeling the GPs knows him or her and the patient trusting the GP) with the GP *before* implementation of ACP and having an ACP conversation?
2. Is there an association between having an ACP conversation and the relationship with the GP *after* implementation of ACP?

**Table 1:** Description of the ACP intervention and summary of the results of the implementation study

**The intervention **[17]**:**
The goal was implementation of ACP in routine (everyday) GP care for older people. The intervention involves a two-step process in which the GP works together with the home care nurse, certified nursing assistant or with the practice nurse to implement ACP. The intervention was delivered by the professionals working in the 12 participating organisations. All the professionals involved were regular care providers of the patients, so no new people were introduced with the delivery of ACP. Health care professionals received amongst others, a training, a manual and conversation aids. The care providers decided which patients they would invite for an ACP discussion. Of course patients did not have to accept the invitation. In addition, conversations could also be initiated by patients themselves. Patients have a first conversation with a home care nurse, a certified nursing assistant or with the practice nurse. Subsequent conversations are with the GP. Our advice to staff was to have one ACP conversation per week to build up experience (we expected this to still be feasible in daily practice). The number of conversations per patient was not determined in advance.
**Results of the implementation study **[17]**:**
Results in the number of ACP conversations and advance directives were modest but positive; ACP conversations were offered to or started with 26% of older patients enlisted with participating organisations. ACP was implemented as routine care. Within respondents who filled in both the pre- and post-test questionnaire (**AQ: correct?**)(*n* = 458), more people had spoken to their GP about hospitalisations (OR 1.66 (1.18 – 2.32)), IC admission (OR 2.12 (1.40 – 3.22)), and treatment preferences in certain circumstances (OR 2.01 (1.42 – 2.84)) after implementation of ACP, compared to before. Advance Directives were drawn up more often (OR 1.54 (1.18—2.00)) after implementation, compared to before.

Based on the literature described, we expect that a better relationship heightens the change of an ACP conversation, and that ACP conversations improve the relationship.

## Methods

### Design and population

Patients aged 75 years or older enlisted with participating organisations filled in questionnaires in a pre-and post evaluation study. ACP was implemented in 10 GP-practices and 2 care homes [17]. Our target group existed of 1) all patients within the participating GP practices aged 75 years or older and 2) all inhabitants of certain wings of the participating care homes. Both the home-dwelling patients and the inhabitants of the care homes all received care from a GP as the main care provider. The Medical Ethics Review Committee of VU University Medical Center has judged that the Medical Research Involving Human Subjects Act (WMO) does not apply to this study and that an official approval of the study by our committee is not required (number 2016.468) because it did not involve any imposing interventions or actions [18]. However, the research team followed main principles of good clinical practice, including e.g. informed consent, confidentiality, risks and benefits assessment, and compliance with protocol [19].

### Procedure and questionnaire

Questionnaires were sent by post to all patients aged 75 years or older within the GP-practice or care home. The first questionnaire was sent before implementation of ACP (February – March 2017), the second was sent approximately 14 months later (April – July 2018). A reminder was sent after 8 to 10 weeks. The questionnaires contained structured questions about experiences with ACP, communication with healthcare providers, relation between respondent and proxy, healthcare status and demographics. They were piloted on a small sample of respondents to ascertain that the questions were clearly formulated and relevant. The participants choosing to respond to the questionnaires implied their consent to participating in the study.

### Questionnaire: ACP

Whether or not an ACP conversation with the GP took place in the 12 months before the pre and post questionnaire, was derived of the following series of questions:

During the last 12 months, have you thought and/or talked about:

- Whether or not you would like to go to the hospital under certain circumstances?
- Whether or not you would like to be admitted to intensive care?
- Whether or not you would like to be admitted to a nursing home?
- Where you would like to die?
- Which treatments you would and would not like to receive in certain circumstances?

For all topics, seven answering options were provided, where it was possible to give more than one answer: a) thought about it, b) talked to my GP about it, c) talked to a doctor (other than the GP), d) talked to another healthcare provider, e) talked to someone else (not a healthcare provider), f) I have recorded it, g) none of these answers.

### Questionnaire: doctor—patient relationship

Data related to the relationship between the patient and his/her GP was collected by means of the following questions:

- How well does your GP know you? Answering options were based on a 4-point Likert scale ( 1 = very well, 4 = badly);
- How much do you trust your GP to provide good care to you in the final stage of life? Answering options were based on a 4-point Likert scale ( 1 = very much trust, 4 = no trust);
- How much do you trust your GP to follow your wishes about medical decisions at the end of your life? Answering options were based on a 4-point Likert scale ( 1 = very much trust, 4 = no trust)

The two questions regarding trust were formulated to specifically target important aspects of ACP conversations. In the analyses to answer question one we used the answers from the first measurement (before implementation), to answer question two we used the difference in answers from both measurements (see data analyses).

### Data analyses

To align the answers before and after implementation, we only used data from patients who filled in both questionnaires. A new variable ‘ACP conversation’ was created, based on the questions on the five topics mentioned above. If the patient had talked to his/her GP on any of the five topics, an ACP conversation has taken place (resulting in a dichotomous variable for ‘ACP conversation’). This was done for the pre and post measurement separately. To answer research question one, the resulting variable from the post measurement was used as dependent variable. To answer research question two, the variable from the post measurement was used as independent variable.

The answers on the three questions about the relationship with the GP were skewed. Therefore, to answer question one, the scores of the questions were recoded into dichotomous variables. For the first 4-point Likert scale question the answering options ‘very well’ and ‘well’ were grouped together and the scores for ‘not so well’ and ‘badly’ were also grouped together. For the other two 4-point Likert scale questions the answering options ‘very much trust’ and ‘fairly much trust’ were grouped together and the scores for ‘not much trust’ and ‘no trust’ were also grouped together.

In our analyses of the data in the pre-post implementation study [17], we saw a relation between ACP conversation and age, marital status, diagnosis of a cardiovascular accident (CVA), no diagnosis, and having a clear idea of future problems one might face. Therefore, we used these characteristics to control for confounding effects in our analyses for research question one. Also, we added ACP conversation at the first measurement (before implementation) as a confounder (we also investigated effect modification, this did not occur). Each model (there were three; one for each relationship variable) used ACP conversation at the end of implementation as the outcome (dependent variable) and the three variables related to the patient-GP relationship before implementation as determinants (independent variables) in multivariable logistic regression.

To answer research question two, the answers to the relationship questions before and after implementation were combined to create three categories:

1. A decrease; for the three variables: knowing the patient less / having less trust in the GP to provide good care at the end of life / having less trust in the GP to follow wishes at the end of life than before.
2. No change in answers before and after implementation.
3. An increase; for the three variables: knowing the patient more / having more trust in the GP to provide good care at the end of life / having more trust in the GP to follow wishes at the end of life than before.

Generalized ordered logistic models were made with the three relationship pre-post-difference variables as outcome and the ACP conversation at the end of implementation as determinant. We used the generalized ordered logistic model because the outcome, being the change in relationship, is ordinal and the generalized model is less restrictive than the proportional odds /parallel lines model [20]. In generalized ordered logistic regression the regression coefficients represent the relationship of the ACP conversation to the odds that a respondent would be in each category or above compared to all lower categories of the relationship variable (in our analyses ‘lower’ versus ‘the same plus more’ and ‘lower plus the same’ versus ‘more’). The model produces a unique set of regression coefficients for each comparison [21]. The data were investigated for potential confounders using Pearsons Chi2 Test, the variable was seen as confounder below a threshold *p*-value of 0.05 (see Additional File, Tables A2-A3-A4). *When confounders were present (only in Table **3**) both crude and adjusted OR’s were presented.* Results are presented as frequencies and odds ratio’s with 95% confidence intervals. All analyses were conducted with Stata SE 14 [22]. The dataset supporting the conclusions of this article is available in the DANS Easy repository [23].

**Table 2:** Characteristics of patients on first measurement who did or did not have an ACP conversation with their GP after implementation of ACP, *n* (%)

	Total (*n* = 458)	No ACP after implementation^a^ (*n* = 308)	ACP after implementation^a^ (*n* = 150)
Age, mean (SD)	80.9 (4.8)	80.2 (4.3)	82.3 (5.5)
Sex, female	286 (63.7)	190 (63.1)	96 (64.9)
Marital status, married	184 (41.1)	134 (44.7)	50 (33.8)
Time living on current address for more than five years	407 (89.5)	277 (90.5)	130 (87.3)
Had an ACP conversation at first measurement (before implementation), yes	101 (22.1)	38 (12.3)	63 (42.0)
My GP knows me …			
- Very well	116 (25.6)	57 (18.8)	59 (39.6)
- Fairly well	272 (60.0)	195 (64.1)	77 (51.7)
- Not so well	56 (12.4)	46 (15.1)	10 (6.7)
- Badly	9 (2.0)	6 (2.0)	3 (2.0)
How much do you trust your GP to provide good care to you in the final stage of life?			
- Very much trust	197 (45.4)	114 (39.6)	83 (56.9)
- Fairly much trust	208 (47.9)	152 (52.8)	56 (38.4)
- Not much trust	26 (6.0)	20 (6.9)	6 (4.1)
- No trust	3 (0.7)	2 (0.7)	1 (0.7)
How much do you trust your GP to follow your wishes about medical decisions at the end of your life?			
- Very much trust	173 (40.7)	94 (33.6)	79 (54.5)
- Fairly much trust	216 (50.8)	160 (57.1)	56 (38.6)
- Not much trust	34 (8.0)	24 (8.6)	10 (6.9)
- No trust	2 (0.5)	2 (0.7)	0

^a^ Missing data: age no-acp *n* = 9 acp *n* = 4; sex no-acp *n* = 7 acp *n* = 2; marital status no-acp *n* = 8 acp *n* = 2; living situation no-acp *n* = 2 acp *n* = 1; GP knows me no-acp *n* = 4 acp *n* = 1; trust good care no-acp *n* = 20 acp *n* = 4; trust follow wishes no-acp *n* = 28 acp *n* = 5

**Table 3:** Association between the patient-GP relationship before implementation and ACP conversations after implementation of ACP

	Patients without ACP conversation after implementation *N* = 308	Patients who had an ACP conversation after implementation *N* = 150	OR (95% CI)	Adjusted OR (95% CI)^a^
My GP knows me well^b^	252 (82.9)	136 (91.3)	**2.16 (1.14 – 4.10)**	1.81 (0.90 – 3.67)
I trust my GP to provide good care to me in the final stage of life^c^	266 (92.4)	139 (95.2)	1.64 (0.68 – 3.94)	1.60 (0.62 – 4.15)
I trust my GP will follow my wishes about medical decisions at the end of your life^d^	254 (90.7)	135 (93.1)	1.38 (0.65 – 2.95)	1.33 (0.56 – 3.15)

^a^ Adjusted for: age, marital status, diagnosis of CVA, no diagnosis, idea future problems, ACP conversations *before* implementation of ACP. Bold indicates *p* < 0.05

^**b**^ Model *n* = 419 due to missing values

^**c**^ Model *n* = 401 due to missing values

^**d**^ Model *n* = 393 due to missing values

**Table 4:** Association between the difference in patient-GP relationship and ACP conversations after implementation of ACP

	Less after implementation n (%)	No change before and after implementation n (%)	More after implementation n (%)	Less versus no change and more ^a^ OR (95% CI)	Less or no change versus more ^a^ OR (95% CI)
	My GP knows me… (*n* = 444)				
● No ACP conversations after implementation	44 (14.7)	200 (66.7)	56 (18.7)	Ref	Ref
● ACP conversation(s) after implementation	22 (15.3)	90 (62.5)	32 (22.2)	0.95 (0.55 – 1.66)	1.24 (0.76 – 2.03)
	I trust the GP will provide good care (*n* = 415)				
● No ACP conversations after implementation	55 (20.0)	178 (64.7)	42 (15.3)	Ref	Ref
● ACP conversation(s) after implementation	11 (7.9)	103 (73.6)	26 (18.6)	**2.93 (1.48 – 5.80)**	1.27 (0.74 – 2.17)
	I trust the GP will follow my wishes (*n* = 405)				
● No ACP conversations after implementation	44 (16.6)	174 (65.7)	47 (17.7)	Ref	Ref
● ACP conversation(s) after implementation	10 (7.1)	97 (69.3)	33 (23.6)	**2.59 (1.26 – 5.32)**	1.43 (0.87 – 2.36)

^a^ Generalized ordered logistic regression. Bold indicates *p* < 0.05

## Results

### Characteristics of patients

In total, 2292 patients received a questionnaire, 458 (20.0%) patients filled in both questionnaires. The mean age of the participants in the study was 80.9 years, the majority (63.7%) was female and married (41.1%) (Table 2). Most patients indicated that their GP knew them fairly well (60.0%) or very well (25.6%). They very much trusted their GP to provide good care in the final stage of their life (45.4%) and to follow their wishes about medical decisions at the end-of-life (40.7%). We refer to our publication on the pre-post implementation study for a non-response analysis [17]. See Additional File Table A1 for ACP conversations and responses to the three relationship-questions before and after implementation of ACP.

## The association between the patient’s relationship with the GP before implementation of ACP and having an ACP conversation during implementation

There is no statistically significant association between a positive answer on the GP knowing the patient and the trust questions and having an ACP conversation (Table 3; adjusted OR).

## The association between having an ACP conversation and the relationship with the GP after implementation of ACP

See Table 4 for a description of the newly generated ‘difference variable’ that compares answers on the relationship questions before and after implementation. No confounders were found with regard to the relationship questions (Additional File, Tables A2, A3 and A4). There is no association between the difference before and after implementation regarding ‘my GP knows me’ and having an ACP conversation during implementation (Table 4). There is an association between the difference before and after implementation regarding the two trust-questions and having an ACP conversation during implementation. People who had an ACP conversation at the end of the implementation period more often indicated that trust remained the same or was higher after implementation, compared to patients who did not have an ACP conversation. So, more simply said, a reduction in trust was less likely to happen to patients who had an ACP conversation compared to patients who did not have an ACP conversation.

## Discussion

We examined the association between having an ACP conversation and the patient’s relationship with the GP before and after the conversation. There was no higher odds of having an ACP conversation for patients who indicated their GP knows them well or who trusted their GP with regard to end-of-life care. However, we found higher odds of unchanged or increased trust in their GP with regard to end-of-life and goal-concordant care after ACP conversations.

### Strengths and limitations of this study

Since other studies on the subject were qualitative, this study is a valuable addition. By comparing answers from the questionnaires before and after implementation, it provides the possibility to take a closer look at the interplay between ACP and the patient-doctor relationship, by examining ACP as the starting point and as the outcome. The large sample allowed us to select patients who answered both the pre and post implementation questionnaire, making our analyses more robust. However, a limitation of the pre/post design is that other factors could have influenced the doctor-patient relationship besides ACP conversations. We had close contact with all participating organisations and by our knowledge they did not implement other interventions, but we cannot rule out that there are other factors at play. Since we did not randomly assign having an ACP discussion or not, we cannot claim causal relations. Also, because the relationship data were skewed, we dichotomised the four point scale, which resulted in a loss of detail. Patients were invited to fill in the questionnaire through a letter from the GP, and therefore results may be biased towards older people who have a good relationship with their GP. Most Dutch older patients (may) have a long term relationship with their GP, and care provision in Dutch GP practices is generally of high quality and characterised by open communication [24, 25]. Results may be different in other countries with another position for GPs within the healthcare system [26]. We do not have information on the depth and scope of the conversations, as the questionnaire had a limited focus on topics. Also, because most questions in the questionnaire were on ACP, people who were less comfortable thinking about future health problems may have been less inclined to respond. For those who were comfortable with the questions, the first questionnaire may have influenced our results on the second measurement, by drawing attention to the possibility of ACP conversations (see below).

### Relationship with GP as starting point for ACP

It has been seen in previous studies that both GPs and patients talk about ACP in light of knowing or not knowing each other. However, this seems mainly related to preferences in writing. According to patients, for preferences to be respected, it is critical to be ‘‘known’’ to a provider [9]. GPs describe that a ‘knowing’ relationship develops over time, and for this regularity of contact and trust is important [27]. Over time GPs got a better understanding of the patient, and this understanding is considered to be sufficient; it reduces the need for preferences to be in writing. For patients the GPs do not know, ACP documents are considered to be useful [27]. Whether a good relationship is a prerequisite for ACP conversations or not, may have implications for the timing of ACP conversations with new patients; is it necessary to know a patient well before you start an ACP conversation, or can it be part of the process of getting to know each other? Our results indicate that the latter may be the case. Studies where ACP conversations are held with persons with no previous connection to the patient, like volunteers or special healthcare agents, can show positive results [28, 29] or have the same results as programs in which ACP is delivered by healthcare providers already known to the patient [30]. That we found no higher odds of conversations for patients who trust their GP might also be explained by the high levels of trust for all patients included in the study; on the first measurement much or very much trust to provide good care was already present in 93.3% of patients and 91.5% already had much or very much trust in the GP to follow their wishes. Every two years the Dutch Health Care Consumer Panel is asked about trust in healthcare, and GPs are among the highest scoring professionals with 89% of patients having trust in GP care in 2020 [31]. Whether trust is a prerequisite for ACP conversations or not becomes superfluous in everyday care if almost all patients trust their GPs.

### Building relationships with ACP

Although we did not find evidence for trust as a prerequisite for ACP conversations, we found higher odds of a unchanged or better doctor-patient relationship after ACP conversations. If the relationship can improve by having ACP conversations, this is an important additional benefit of having them. In our study the effect was mainly seen in patients with a *decrease* in trust in the GP to provide good care (20% of patients with no ACP conversation versus 8% of patients with an ACP conversation) and a *decrease* in trust in the GP to follow the patients’ wishes (17% of patients with no ACP conversation versus 7% of patients with an ACP conversation), instead of an *increase* in trust. Maybe patients were expecting an ACP conversation would be offered to them by the GP after receiving the first questionnaire, and some may have been disappointed when this did not happen. It has been shown that when patients expect an ACP conversation but their concerns are not addressed by the doctor, this threatened the relationship [10]. However, that we did not detect a rise in trust may also be related to both the aforementioned ceiling effect and the size of the study sample. Tentatively it may be said that engaging in ACP may strengthen or improve the doctor-patient relationship. A study among nurses in long-term care facilities also found that the relationships with the residents and their family developed and strengthened after implementation of ACP [32]. People may be less inclined to ACP if they don’t trust their healthcare provider to follow their wishes [16] or with healthcare providers who did not show interest in them before (other than with regard to ‘medical’ topics) [8], but during conversations this may change. Maybe the issue is not so much whether or not there is an ongoing relationship before you start ACP, but whether the healthcare provider provides an opportunity to discuss worries and fears by offering an ACP conversation, and shows compassion, uses communication strategies and has conversation skills during ACP. In that respect, the emphasis that is sometimes placed on Advance Directives or quality assurance (e.g. how many patients have had a conversation) can have detrimental effects by shifting the focus away from the person-centered approach [33].

## Conclusion

A good relationship does not seem to be a prerequisite for ACP conversations, but by having these conversations the doctor-patient relationship may strengthen or improve. This information can be used when motivating GPs to take up ACP conversations with their older patients. Future studies should aim to disentangle the complex relation between trust and ACP, e.g. by a qualitative approach with a longitudinal qualitative study following patients and GPs in the ACP process or by a quantitative approach with Structural Equation Modelling.

## Supplementary Information

**Additional file 1: Table A1.** ACP conversations and responses to the three relationship-questions before and after implementation of ACP. **Table A2.** Characteristics of patients and the difference before and after implementation in answers to ‘how well does your GP know you’. **Table A3.** Characteristics of patients and the difference before and after implementation in answers regarding trust in their GP to provide good care in the final stage of life. **Table A4.** Characteristics of patients and the difference before and after implementation in answers regarding trust in their GP to follow their wishes about medical decisions in the final stage of life

## Data Availability

The dataset supporting the conclusions of this article is available in the DANS Easy repository, https://doi.org/10.17026/dans-z9r-2hbz.

## Abbreviations

ACP: Advance care planning
CI: Confidence intervals
CVA: Cardiovascular accident
GP: General practitioner
OR: Odds ratio
