# Supplementary material for: The patient’s relationship with the General Practitioner before and after Advance Care Planning: pre/post-implementation study
Source: BMC Geriatr. 2022 Jul 5;22:558. doi: 10.1186/s12877-022-03256-4 (PMC9254656; doi:10.1186/s12877-022-03256-4)
Supplement: Supplementary file 1 — Additional file 1: Table A1. ACP conversations and responses to the three relationship-questions before and after implementation of ACP. Table A2. Characteristics of patients and the difference before and after implementation in answers to ‘how well does your GP know you’. Table A3. Characteristics of patients and the difference before and after implementation in answers regarding trust in their GP to provide good care in the final stage of life. Table A4. Characteristics of patients and the difference before and after implementation in answers regarding trust in their GP to follow their wishes about medical decisions in the final stage of life [file 12877_2022_3256_MOESM1_ESM.docx]

***The patient’s relationship with the General Practitioner before and after Advance Care Planning: pre/post-implementation study***

**Supplementary data.**

**Table of contents:**

- Table A1. ACP conversations and responses to the three relationship-questions before and after implementation of ACP
- Table A2. Characteristics of patients and the difference before and after implementation in answers to ‘how well does your GP know you’
- Table A3. Characteristics of patients and the difference before and after implementation in answers regarding trust in their GP to provide good care in the final stage of life
- Table A4. Characteristics of patients and the difference before and after implementation in answers regarding trust in their GP to follow their wishes about medical decisions in the final stage of life

**Table A1. ACP conversations and responses to the three relationship-questions before and after implementation of ACP**

|  | Pre measurement  (n=458) ^a^ | Post measurement  (n=458) ^a^ |
| --- | --- | --- |
| ACP conversation, yes | 101 (22.1) | 150 (32.8) |
| My GP knows me … |  |  |
| - Very well | 116 (25.6) | 128 (28.5) |
| - Fairly well | 272 (60.0) | 269 (59.9) |
| - Not so well | 56 (12.4) | 47 (10.5) |
| - Badly | 9 (2.0) | 5 (1.1) |
| How much do you trust your GP to provide good care to you in the final stage of life? |  |  |
| - Very much trust | 197 (45.4) | 201 (46.3) |
| - Fairly much trust | 208 (47.9) | 208 (47.9) |
| - Not much trust | 26 (6.0) | 23 (5.3) |
| - No trust | 3 (0.7) | 2 (0.5) |
| How much do you trust your GP to follow your wishes about medical decisions at the end of your life? |  |  |
| - Very much trust | 173 (40.7) | 196 (45.6) |
| - Fairly much trust | 216 (50.8) | 204 (47.4) |
| - Not much trust | 34 (8.0) | 25 (5.8) |
| - No trust | 2 (0.5) | 5 (1.2) |

^a^ Missing data: My GP knows me pre n=5 post n=9; I trust my GP to provide good care pre n=24 post n=24; I trust my GP to follow my wishes pre n=33 post n=28.

**Table A2. Characteristics of patients and the difference before and after implementation in answers to ‘how well does your GP know you’**

|  | Total (n=444) ^1^ | My GP knows me; less well after implementation (n=66) | No change (n=290) | My GP knows me; better after implementation (n=88) | Pearson chi2, p-value |
| --- | --- | --- | --- | --- | --- |
| Age, mean (SD) | 82.2 (4.9) | 82.3 (4.9) | 82.1 (4.9) | 82.2 (5.0) | 0.97 (Anova) |
| Sex, female | 278 (63.8) | 44 (68.8) | 176 (62.0) | 58 (65.9) | 0.53 |
| Marital status, married | 167 (38.4) | 27 (42.2) | 104 (36.8) | 36 (40.9) | 0.62 |
| Time living on current address for more than five years | 404 (91.4) | 61 (92.4) | 262 (90.7) | 81 (93.1) | 0.74 |
| At least one diagnosis (vs none) | 323 (77.6) | 53 (84.1) | 203 (75.2) | 67 (80.7) | 0.23 |
| Do you have a clear idea about future health problems that you might face; yes | 176 (39.9) | 23 (34.9) | 116 (40.3) | 37 (42.5) | 0.62 |

^1^ Characteristics as measured at the second measurement (after implementation). Missing data: my GP knows me n=14 out of 458; age n=13; sex n=8; marital status n=9; living situation n=2; diagnosis (dichotomous) n=28; idea future problems n=3

**Table A3. Characteristics of patients and the difference before and after implementation in answers regarding trust in their GP to provide good care in the final stage of life**

|  | Total (n=415) ^1^ | I trust my GP to provide good care; less after implementation (n=66) | No change (n=281) | I trust my GP to provide good care; more after implementation (n=68) | Pearson chi2, p-value |
| --- | --- | --- | --- | --- | --- |
| Age, mean (SD) | 82.1 (4.9) | 81.8 (4.1) | 81.8 (5.0) | 83.3 (5.4) | 0.07 (Anova) |
| Sex, female | 253 (62.0) | 39 (60.9) | 171 (61.5) | 43 (65.2) | 0.85 |
| Marital status, married | 161 (39.6) | 27 (42.2) | 112 (40.4) | 22 (33.3) | 0.51 |
| Time living on current address for more than five years | 372 (91.0) | 57 (86.4) | 253 (91.3) | 62 (93.9) | 0.29 |
| At least one diagnosis (vs none) | 300 (77.7) | 51 (82.3) | 202 (76.8) | 47 (77.1) | 0.64 |
| Do you have a clear idea about future health problems that you might face; yes | 172 (42.2) | 24 (36.4) | 124 (44.9) | 24 (36.4) | 0.26 |

^1^ Missing data: my GP knows me n=43 out of 458; age n=11; sex n=7; marital status n=8; living situation n=6; diagnosis (dichotomous) n=29; idea future problems n=7

**Table A4. Characteristics of patients and the difference before and after implementation in answers regarding trust in their GP to follow their wishes about medical decisions in the final stage of life**

|  | Total (n=405) ^1^ | I trust my GP to follow my wishes; less after implementation (n=54) | No change (n=271) | I trust my GP to follow my wishes; more after implementation (n=80) | Pearson chi2, p-value |
| --- | --- | --- | --- | --- | --- |
| Age, mean (SD) | 82.0 (4.9) | 82.1 (4.7) | 81.8 (4.7) | 82.6 (5.6) | 0.45 (Anova) |
| Sex, female | 245 (61.6) | 29 (53.7) | 161 (60.8) | 55 (69.6) | 0.16 |
| Marital status, married | 158 (39.8) | 22 (40.7) | 101 (38.3) | 35 (44.3) | 0.62 |
| Time living on current address for more than five years | 362 (90.7) | 48 (88.9) | 243 (90.7) | 71 (92.2) | 0.81 |
| At least one diagnosis (vs none) | 290 (76.7) | 43 (82.7) | 196 (76.9) | 51 (71.8) | 0.37 |
| Do you have a clear idea about future health problems that you might face; yes | 168 (42.2) | 18 (33.3) | 121 (45.5) | 29 (37.2) | 0.16 |

^1^ Missing data: I trust my GP to follow my wishes n=53 of 458; age no-trust n=1 trust n=10; sex n=7; marital status n=8; living situation n=6; diagnosis (dichotomous) n=27; idea future problems n=7
